# Resident resource use in clinical settings: an institutional survey

**DOI:** 10.5195/jmla.2026.2396

**Published:** 2026-10-01

**Authors:** Nell Aronoff, Laurel Mueller

**Affiliations:** 1 naronoff@buffalo.edu, Associate Librarian and Liaison to the Jacobs School of Medicine and Biomedical Sciences, University Libraries, University at Buffalo, Buffalo, NY; 2 darlingm@buffalo.edu, Medical and Biomedical Sciences Liaison Librarian, University Libraries, University at Buffalo, Buffalo, NY

**Keywords:** Graduate medical education, internship and residency, medical residents, medical libraries, surveys and questionnaires, resource use, clinical decision making

## Abstract

**Background:**

We explored which point of care (POC) resources medical residents at the University at Buffalo use in clinical settings. We specifically sought to gather feedback from residents who help train medical students to see how they discover, use, and promote resources to support patient care.

**Case Presentation:**

We developed an eight question Qualtrics survey. Residency program coordinators emailed the survey to approximately 445 residents in the following programs: Family Medicine, Internal Medicine, Combined Internal Medicine and Pediatrics, Neurology, Child Neurology, Obstetrics and Gynecology, Pediatrics, Psychiatry, Surgery, Plastic Surgery, and Vascular Surgery. The programs we targeted all correspond to required clerkship rotations. The survey was open for two weeks in June 2025. Thirty-nine residents (8.8%) completed our survey. Thirty-five, or nearly 90% of respondents, reported that they frequently use UpToDate, followed by twenty-three (59.0%) that use OpenEvidence, and seventeen that use MDCalc (43.6%). The residents we surveyed were inclined to recommend similar resources to medical students. They most often hear about new resources from other residents and attendings, via announcements on social media or podcasts, or by attending grand rounds and conferences.

**Conclusions:**

Our results highlight the continued popularity of UpToDate among medical trainees but also suggest that residents frequently use OpenEvidence in clinical settings. Further studies are needed to determine the impact of artificial intelligence tools on patient care and learning outcomes.

## BACKGROUND

The Accreditation Council for Graduate Medical Education (ACGME) Common Program Requirements for Residency Programs includes proficiencies in the acquisition and application of Medical Knowledge (4.6) and Practice-Based Learning and Improvement (4.7) [1].

In addition to the ACGME requirements, the Association of American Medical Colleges’ thirteen core Entrustable Professional Activities (EPAs) for graduating medical students includes the ability to “form clinical questions and retrieve evidence to advance patient care” [2]. Based on these expectations, residents must develop the ability to recognize when a clinical scenario calls for information and identify where to find the answers to their clinical questions.

Providers use POC resources to educate themselves and others, including patients and learners, and to make clinical decisions [3]. Previous studies have looked at where students, residents, and other clinicians find information to support patient care. They illustrate the enduring popularity of UpToDate, PubMed, the Cochrane Library, and drug information sources, but it is important to note that they were all published before generative AI tools were readily available [4–10]. For example, Blake and Tong surveyed Pediatrics, Pediatric Infectious Disease, Trauma, and Family Medicine residents after a month-long rotation and found that they relied most heavily on UpToDate, PubMed, and LexiComp [4]. Brassil et al.’s survey of practitioners at Baystate Medical Center found that 80% of respondents used UpToDate and 65% used PubMed, followed by Epocrates, Micromedex, Google Scholar, and Cochrane [5]. Petersen, Earl, and Wilson’s review of library usage trends over a sixteen-year period at the University of Tennessee showed that residents and fellows consistently used UpToDate, PubMed, and the Cochrane Library [9].

Part of the challenge of determining what resources to use is that there are more options than ever before. Bernstein et al.’s survey of 662 internal medicine residents in the US revealed that residents used an average of seven traditional resources and two novel resources [11]. Traditional resources included broad categories like digital clinical resources (i.e., UpToDate), clinical experience, journal articles, textbooks, and guidelines. Novel resources included blogs, podcasts, and YouTube videos. Cook et al. suggest that a lack of awareness about where to search and the exponential amount of information physicians must wade through are barriers to POC learning [12]. Along with the exponential increase in medical information, there has also been an influx of new tools and resources. This so-called tyranny of choice may overwhelm residents and lead them to return to familiar tools even if better resources exist [13].

The use of artificial intelligence (AI) has exploded in the past few years, further complicating where residents should turn when deciding where to search for clinical information. Some AI tools are easier to access, easier to use, or more reliable than others. As of this writing, ChatGPT is perhaps the best-known AI chatbot. As Lahat and colleagues state, while ChatGPT has the potential to improve patient outcomes, it must be used in tandem with human knowledge [14]. A more recent addition to the AI landscape is OpenEvidence, an AI chatbot trained on content from JAMA and The New England Journal of Medicine. As more and better AI tools are available, clinicians will find new ways to address POC questions.

## CASE PRESENTATION

### Institutional Context

The University at Buffalo (UB) is a large public research institution. UB has over sixty resident and fellow training programs with over 800 residents and fellows [15]. Until August 2024, there was one primary library liaison to the Jacobs School of Medicine and Biomedical Sciences. At that time, a second library liaison was hired to support the school which has faculty, staff, undergraduate, graduate, medical, and PhD students, residency, and fellowship programs, and twenty-six basic science and clinical departments. Since there is no dedicated university teaching hospital, residents are located at nine affiliated hospitals and in various UBMD clinics across Western New York, leading to limited interactions between residents and their assigned liaison librarians [16]. Librarians have interacted with residents at new resident orientations and have been invited to present during grand rounds and academic half day sessions. Some hospital partners provide their own library resources and services, which means that residents may also be receiving assistance from full time or circuit hospital librarians.

### Purpose

We sought to explore which POC resources medical residents at our institution use in clinical settings.

Exploratory questions that guided our survey design included: 1) Are they still relying heavily on UpToDate?; 2) What changes have there been since previous studies?; and 3) Have the emergence of artificial intelligence tools changed their workflows?

### Survey Population

We sought to query residents that regularly interact with medical students in required clerkships to learn how residents discover and promote resources to support patient care. The core clerkships consisted of Family Medicine, Medicine, Advanced Medicine, Neurology, Obstetrics and Gynecology, Pediatrics, Psychiatry, and Surgery so we targeted residents in the following programs: Family Medicine, Internal Medicine, Combined Internal Medicine and Pediatrics, Neurology, Child Neurology, Obstetrics and Gynecology, Pediatrics, Psychiatry, Surgery, Plastic Surgery, and Vascular Surgery. This amounted to approximately 445 residents.

### Survey Instrument and Distribution

Our protocol was approved by the UB Institutional Review Board in May 2025. We shared a draft of the survey with seven residents for feedback about how to bolster the response rate. They suggested that residency program coordinators email the survey on our behalf and mentioned that a survey incentive could help. Another resident suggested that we could promote the survey during grand rounds or academic half days; however, we could not schedule visits with every program during the survey period. We were also concerned that promoting the survey to certain programs would result in response bias.

The final Qualtrics survey instrument contained eight questions. The full survey is available in Appendix A. Given the demands on potential respondents’ time, we intended for the survey to take less than ten minutes to complete. Program coordinators emailed the survey twice during a two-week period in June 2025. We offered three $50 gift cards as an incentive to participate.

### Response Rate and Respondents’ Program Demographics

Using resident lists from Graduate Medical Education (GME) program websites, we estimated there were 445 residents across the eleven programs affiliated with required medical student clerkships in academic year 2024–2025. Forty medical residents responded to the survey. One response was disregarded from analysis because they did not provide any usable information besides their program name. With 39 completed responses, there was an 8.8% response rate. Due to the low response rate, we were also unable to provide in-depth analysis of specialty-specific resource use.

The survey began with one demographic question that asked participants to identify their program affiliation. The resident response breakdown is as follows: Internal Medicine (n=16, 41%); Family Medicine (n=6, 15.4%); Pediatrics (n=6, 15.4%); Neurology or Child Neurology (n=4, 10.3%); Obstetrics/Gynecology (n=4, 10.3%); Surgery, Plastic Surgery, or Vascular Surgery (n=2, 5.1%); and Psychiatry (n=1, 2.6%).

### Resources Used for Clinical Decision Support

Next, we asked respondents what information resources they use most frequently to answer clinical questions (Figure 1). Frequency was defined as resources they engage with on a daily or weekly basis, and information resources were defined (but not limited to) medical apps, websites, databases, calculators, textbooks, and AI tools. This was an open-ended question with no set limits on the number of responses to include, and respondents were not offered a list of resources to select from to reduce response bias.

**Figure 1:**
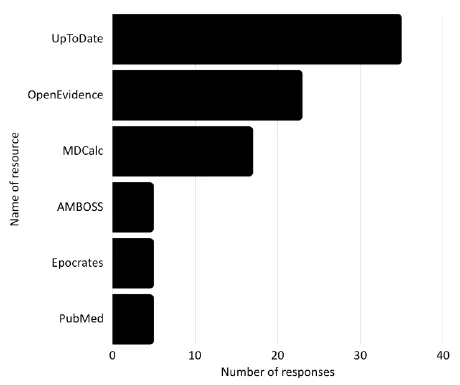
Top resources used frequently by residents for clinical decision support

Nearly all survey takers listed two or more tools. In total, 136 tools were listed among the thirty-nine respondents. This is an average of 3.5 resources per resident at our institution, with a population standard deviation of 1.6. Four respondents (10.3%) only consulted UpToDate for clinical decision-making. The other 35 residents (89.7%) reported using more than one resource. Thirty-five respondents (89.7%) used UpToDate for clinical decision support. Over half of the respondents (n=23, 59.0%) frequently consulted OpenEvidence, the AI-powered medical support platform. One other AI platform, ChatGPT, was used frequently by three participants (7.7%). The third most referenced tool used for clinical decision support was MDCalc (n=17, 43.6%). AMBOSS, Epocrates, and PubMed were each mentioned by 5 (12.8%) respondents. Four participants (10.6%) consulted the CDC vaccine schedule and Lexidrug. Guidelines from professional associations like the American College of Obstetricians and Gynecologists and the Infectious Disease Society of America, as well as general or specialty medical journals like JAMA and Obstetrics & Gynecology were listed by 3 (7.7%) or fewer people. One respondent (2.6%) mentioned Google, and another participant listed Google Scholar. Participants responded with 36 unique POC resources, ten of which are available through the UB Libraries. See Appendix B for a full list of resources.

To understand how residents support medical students’ clinical education through resource sharing, participants were asked an open-ended question about what resources they recommend to medical students during their required clerkships (Figure 2). Notably, the question did not define or limit responses to tools only used for clinical-decision support, so respondents could reference any resources that would help medical students in their educational and clinical training. Residents listed similar resources to the ones they consulted for their own use.

**Figure 2:**
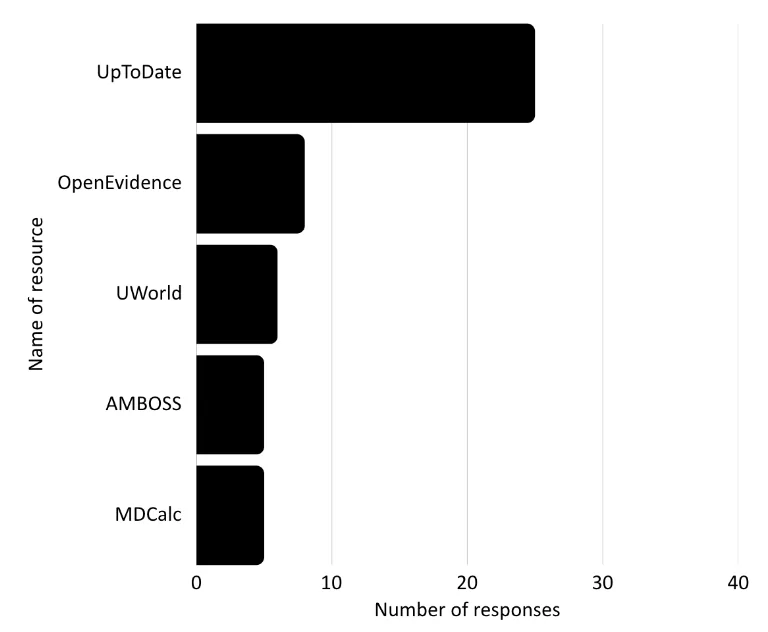
Top resources shared by residents with medical students during clerkships

UpToDate was again listed as the top resource (n=25, 64.1%), and OpenEvidence was the second most suggested resource (n=8, 20.5%). Respondents recommended test preparation materials, such as UWorld and AMBOSS. Survey takers also indicated specialty-specific textbooks, YouTube videos, and podcasts to support medical student learning and training. See Appendix C for a full list of resources.

### Learning about New Resources

To explore how residents learn about new resources, one survey question asked residents who tells them about new resources. Regarding the who, residents were given seven choices as well as an “other” option. Participants could select all that apply. Nearly all the respondents (n=36, 92.3%) reported learning about new resources from other residents. Attendings were also frequently selected as a source for tool sharing (n=29, 74.4%), followed by medical students (n=10, 25.6%), other healthcare professionals such as nurses and administrators (n=4, 10.3%), and librarians (n=4, 10.3%). One respondent filled out the write-in option and listed “Internet” (n=2.6%).

In conversation with the previous inquiry, respondents were asked to identify where they find out about new resources. They were given a list of options and an “other” write-in box and asked to check all that apply. Almost half of participants said that they used social media to learn about new resources (n=18, 46.2%). Other top selections included podcasts (n=17, 43.6%), grand rounds (n=16, 41%), conferences (n=15, 38.5%), journal articles (n=12, 30.8%), and professional associations (n=9, 23.1%). One person wrote in “Almost always word of mouth.” One participant (2.6%) selected library guides.

To determine how residents accessed fee-based resources and gauge if they were purchasing resources themselves, we asked residents to report on how they accessed subscription-based clinical resources. Two-thirds of respondents used hospital subscriptions to access paid resources (n=26, 66.7%), nearly equal to those who utilized them through the UB Libraries (n=25, 64.1%). Six residents bought their own (15.4%). Four respondents (10.3%) indicated “other.” Two respondents left the write-in blank, one resident wrote “n/a,” and another indicated they only use free options.

The last question invited respondents to relay anything else about their clinical resource use they wanted to share with their liaison librarians. Nine residents opted to provide additional information. Several residents highlighted the growing reliance on OpenEvidence to support clinical reasoning. Access limitations were also mentioned, including issues using resources off-site at home addresses, along with financial restrictions like having to pay up front for professional association memberships and board studying materials before being reimbursed by a program. Another person explained that the usefulness of each resource depends on the learner and the situation based on the degree of diagnostic or clinical uncertainty.

## DISCUSSION

We sought to determine what resources medical residents at our institution without a designated teaching hospital consult on a daily or weekly basis for clinical decision making. Residents at our institution reported using around 3.5 resources on a frequent basis. This is lower than the results from a nationwide survey of internal medicine residents, which found that residents consulted an average of nine resources for POC and general medical information [11]. One explanation for this is that we did not inquire about what resources residents used to support their education and training or research endeavors.

Results showed that residents continue to rely on UpToDate in clinical settings. According to one respondent, “UpToDate is the most important.” Preference for UpToDate is consistent with other articles that studied resident resource use [5–10]. While UpToDate was the most frequently listed tool in survey responses, four residents did not engage with the platform on a regular basis. One resident noted UpToDate is useful for its high-quality summaries when physicians are more certain of their clinical diagnosis but explained the product “loses its helpfulness when your certainty of the culprit diagnosis is low.”

Though we did not provide a pre-determined list of resources, similar trends appeared in medical resident responses across programs. Notably, residents cited medical specialty-specific professional associations, journals, textbooks, and podcasts as frequently consulted resources. Residents are relying on these tools when in search of specialized information. While the low response rate prevented us from performing further sub-analysis on programmatic resource use, other studies have addressed resource use among residents in specific programs [6, 11, 17].

Recent research illustrates that medical students and professionals use popular websites like Google and Wikipedia as well as databases including PubMed, Medline, and Google Scholar to answer clinical questions [5, 6, 8–10]. In Duran Nelson et al.’s study on resource use among internal medicine residents at POC, researchers found that residents were more distrustful of information provided by Google and Wikipedia but engage with these resources during the “discovery phase” before searching for additional information through a trusted source [6]. Our results show less frequent reliance on these tools; despite the efficiency and public accessibility these tools offer. The shift could be due to evolving clinical decision support technologies or a greater emphasis in medical and resident education on using high-quality evidence-based sources.

Residents are increasingly relying on AI-powered tools in clinical settings. As demonstrated by our results, residents have begun to identify AI tools like OpenEvidence as among their most frequently consulted POC platforms. While we did not include questions on how residents determine which tools to use in medical decision making, previous scholarship on resource use among medical professionals found speed and ease of use were paramount [6, 10, 18]. Residents seek efficient means to locate high-quality, evidence-based medical information to answer clinical questions. Because AI tools synthesize large amounts of information almost instantaneously, they often reduce the time needed to search within individual databases or journals. This suggests that AI tools may continue to gain popularity among medical trainees.

Communication among residents, attendings, and medical students is essential for resource sharing, as residents are learning about new resources from other medical professionals. Brassil et al. found communication with colleagues was one of the most popular methods in finding an answer for a clinical question, so it follows that residents would learn about new resources most often from their peers [5]. As to where residents are learning about new resources, they discover new tools in academic and online contexts. Bernstein et al. found internal medicine residents are relying more on audio-visual media, including video streaming platforms and podcasts, for general and POC medical information compared to earlier studies [11]. Specialty-specific podcasts were included as frequently used resources among residents, and these podcasts, along with the video streaming platform YouTube, were recommendations residents shared with medical students during their required clinical rotations.

One of our goals was to assess how residents are accessing resources and if residents are paying out of pocket for subscription-based resources. Most residents utilize subscription-based tools provided by the UB Libraries or through their hospital affiliations. Less than one-fifth of respondents reported paying for subscription-based sources themselves. A remaining question is whether residents are finding what they need for free or through their university or hospital connections, or whether they are going without other beneficial tools because they can’t afford to purchase them.

### Implications for Practice

Data gathered from the survey has informed how we meet the information needs of residents at our institution. Though over half of the respondents disclosed that they access clinical resources through the UB Libraries, very few residents learned about new tools from librarians or via library guides. This suggests that we could be better at promoting our subscription resources to this dispersed user group, and further publicizing efforts can occur through instruction sessions and presentations given to residents. As an example, while we provide VisualDx, it was not mentioned as a frequently used resource.

Given the growing popularity of OpenEvidence for POC information, we contacted OpenEvidence customer service to provide us with unlimited access to the platform since it is currently only available to verified healthcare professionals with a National Provider Identifier (NPI). We hope expanded access will allow us to incorporate it into resident instruction sessions, while also highlighting current POC library subscriptions. We have adjusted literature searching lesson plans to demonstrate other AI-powered tools, like the scientific literature searching platform Consensus, to address interest in AI products for faster literature searching. Results from this study will impact resources listed on our libraries’ clinical resources and AI research guides. As usage and reliance on AI products increases, librarians may consider surveying their users to better meet user needs and make collection development decisions.

## LIMITATIONS

There were several limitations to the study. Despite our efforts, the survey response rate was low, representing less than one tenth of the total population to which the survey was distributed. Therefore, we are unable to assess the full scope of resources residents consult for clinical care. Alternate time frames and expanding promotional efforts may have helped increase the response rate.

Offering open-ended responses for questions rather than providing a list of resources reduced response bias. However, some respondents listed general publication types, like review papers or case files, rather than identifying specific products they use to access medical information for clinical decision support. Inputting resources manually may have led to residents listing fewer resources than they actually use.

Finally, although this was outside the scope of our study, we did not investigate how residents are using these tools to make clinical decisions or how they appraise and integrate evidence to promote better patient care. The impact that the resources have on health outcomes is a separate matter that has been studied by Marshall and others [8, 19, 20] but would be worth pursuing again considering the changing information landscape.

## CONCLUSION

Our findings suggest that medical residents at UB tend to rely on a few select tools that they hear about through other residents and attending physicians. Prior studies have also demonstrated a preference for UpToDate, but our results indicate that AI tools are becoming more popular in this cohort. Further investigations should consider how medical residents are integrating AI resources in their clinical decision-making process alongside more traditional tools like UpToDate and MDCalc. We posit that technological developments offering more efficient access to high-quality information will continue to drive information-seeking behavior among medical residents.

## Data Availability

Data associated with this article are available in the Open Science Framework at https://doi.org/10.17605/OSF.IO/RS98Y.

## SUPPLEMENTAL FILES

**Appendix A:** Resident Resource Use Survey

**Appendix B:** Summary of Resources Used for Clinical Decision Support

**Appendix C:** Summary of Resources Shared with Medical Students
