# Supplementary material for: Resident resource use in clinical settings: an institutional survey
Source: J Med Libr Assoc. 2026 Oct 14;114(4):445–51. doi: 10.5195/jmla.2026.2396 (PMC13670215; doi:10.5195/jmla.2026.2396)
Supplement: Supplementary file 1 [file jmla-114-4-445-s01.pdf]

Appendix A:

Resident Resource Use

Start of Block: Default Question Block

Introduction Welcome to our survey aimed at determining which resources you use in clinical settings. It should take approximately 10 minutes to complete. We appreciate your participation.

Question 1. Select your residency program.

- ○ Family Medicine (1)
- ○ Internal Medicine (2)
- ○ Neurology or Child Neurology (3)
- ○ Obstetrics/Gynecology (4)
- ○ Surgery, Plastic Surgery, or Vascular Surgery (5)
- ○ Pediatrics (6)
- ○ Psychiatry (7)

Question 2. What information resources (medical apps, websites, databases, calculators, textbooks, AI tools) do you use the most frequently to answer clinical questions? Please be specific and list resources you use on a daily or weekly basis.

____

____

____

____

____

Question 3. Who tells you about new resources? Select all that apply.

- ☐ Attendings (1)
- ☐ Other residents (2)
- ☐ Medical students (3)
- ☐ Other healthcare professionals (nurses, administrators, etc.) (4)
- ☐ Librarians (5)
- ☐ Other (7) ____

Question 4. Where do you find out about new resources? Select all that apply.

- ☐ Social media (X, Facebook, TikTok, Instagram, Reddit, etc.) (1)
- ☐ Podcasts (2)
- ☐ Conferences (3)
- ☐ Professional associations (AMA, etc.) (4)
- ☐ Journal articles (5)
- ☐ Grand rounds (6)
- ☐ Library guides (7)
- ☐ Other (9) ____

Question 5. What resources do you recommend to medical students during their clerkships?

____

____

____

____

____

Question 6. How do you access subscription-based (non-free) clinical resources? Select all that apply.

- ▢ Through the UB Libraries (1)
- ▢ Through the hospital (2)
- ▢ I buy my own (3)
- ▢ Other (4) ________________________________

Question 7. Is there anything else you would like to share with us about your clinical resource use?

____

____

____

____

____

Question 8. Would you like to be entered in a raffle to win a $50 Wegmans gift card? If you click yes, you will be taken to a separate survey.

- ◯ Yes (1)
- ◯ No (2)

End of Block: Default Question Block
