# Supplementary material for: Resident resource use in clinical settings: an institutional survey
Source: J Med Libr Assoc. 2026 Oct 14;114(4):445–51. doi: 10.5195/jmla.2026.2396 (PMC13670215; doi:10.5195/jmla.2026.2396)
Supplement: Supplementary file 2 [file jmla-114-4-445-s02.pdf]

Appendix B: Summary of resources used for clinical decision support

Number of Users	Resource	Percentage
35	UpToDate*	89.7%
23	OpenEvidence	59%
17	MDCalc	43.6%
5	AMBOSS	12.8%
5	Epocrates	12.8%
5	PubMed*	12.8%
4	Center of Disease Control (CDC)	10.2%
4	Lexicomp/Lexidrug*	10.2%
3	American College of Obstetricians and Gynecologists (ACOG)	7.7%
3	ChatGPT	7.7%
2	American Society for Colposcopy and Cervical Pathology (ASCCP)	5.1%
2	Infectious Diseases Society of America (IDSA) guidelines	5.1%
2	Journal of the American Medical Association (JAMA)*	5.1%
2	New England Journal of Medicine (NEJM)*	5.1%
2	United States Preventive Services Task Force (USPSTF)	5.1%
2	University at Buffalo Libraries*	5.1%
1	American Academy of Neurology (AAN) apps	2.6%
1	American Academy of Family Physicians (AAFP)	2.6%
1	American College of Cardiology (ACC)/American Heart Association guidelines	2.6%
1	American Diabetes Association (ADA) Guidelines	2.6%
1	Anki	2.6%
1	Children’s Hospital of Philadelphia (CHOP) Clinical Pathways Library	2.6%
1	ClinicalKey*	2.6%
1	Core IM Podcast	2.6%
1	Curbsiders IM Podcast	2.6%
1	Diagnosaurus*	2.6%

1	Google	2.6%
1	Google Scholar	2.6%
1	Obstetrics & Gynecology (The Green Journal)*	2.6%
1	J Drive	2.6%
1	Johns Hopkins	2.6%
1	Prescriber's Guide: Stahl's Essential Psychopharmacology*	2.6%
1	Surgical Council on Resident Education (SCORE) General Surgery Resident Curriculum Portal	2.6%
1	TrueLearn	2.6%
1	Unviersity at Buffalo Pediatrics (UBPeds)	2.6%
1	UWorld Medical Library	2.6%

* Accessible through the University at Buffalo Libraries.
