# Supplementary material for: Resident resource use in clinical settings: an institutional survey
Source: J Med Libr Assoc. 2026 Oct 14;114(4):445–51. doi: 10.5195/jmla.2026.2396 (PMC13670215; doi:10.5195/jmla.2026.2396)
Supplement: Supplementary file 3 [file jmla-114-4-445-s03.pdf]

Appendix C: Summary of resources shared with medical students

Number of Users	Resource	Percentage
25	UpToDate	64.1%
8	OpenEvidence	20.5%
6	UWorld	15.4%
5	AMBOSS	12.8%
5	MDCalc	12.8%
3	Epocrates	7.7%
3	YouTube	7.7%
2	Center of Disease Control (CDC)	5.1%
2	PubMed	5.1%
2	Question Banks	5.1%
1	Anki	2.6%
1	American College of Obstetricians and Gynecologists (ACOG)	2.6%
1	Association of Professors of Gynecology and Obstetrics (APGO) website	2.6%
1	Case Files	2.6%
1	Clinical Neurology and Neuroanatomy: A Localization-Based Approach	2.6%
1	ChatGPT	2.6%
1	COMBANK (TrueLearn)	2.6%
1	Comprehensive Handbook Obstetrics and Gynecology	2.6%
1	Core IM Podcast	2.6%
1	Curbsiders IM Podcast	2.6%
1	Divine Podcast	2.6%
1	DynaMed	2.6%
1	Review Papers	2.6%
1	Sketchy	2.6%
1	Surgery: A Case Based Clinical Review	2.6%
1	UB Libraries	2.6%
1	United States Preventive Services Task Force (USPSTF)	2.6%
